# Predicting the intention and adoption of hydroponic farming among Chinese urbanites

**DOI:** 10.1016/j.heliyon.2023.e14420

**Published:** 2023-03-09

**Authors:** Abdullah Al Mamun, Farzana Naznen, Gao Jingzu, Qing Yang

**Affiliations:** a UKM - Graduate School of Business, Universiti Kebangsaan Malaysia, 43600, UKM Bangi, Malaysia; b UCSI Graduate Business School, UCSI University, Malaysia. Cheras, 56000 Kuala Lumpur, Malaysia

**Keywords:** Hydroponic farming, Urban farming, Knowledge-attitude-behaviour theory, Theory of planned behaviour, China

## Abstract

Urban agriculture has become increasingly popular as a viable solution to the global food insecurity, but the uncontrollable expansion of cities and populations has led to the significant decrease in cultivable space for conventional soil-based farming. Unlike conventional agriculture, soil-less hydroponic farming allows sustainable food production in urban areas without compromising the environment and public health. Using both theory of planned behaviour and knowledge-attitude-behaviour theory with four additional factors, the current study examined the intention and adoption of hydroponic farming among Chinese urbanites. An online survey, which involved 661 respondents from different cities in China, was conducted. The results of partial least squares structural equation modeling revealed the significant influence of tolerance of diversity, innovativeness, and knowledge about hydroponic system on attitude towards hydroponic system. Additionally, knowledge and attitude towards hydroponic system, perceived need for hydroponic farming, and hydroponic farming compatibility were found to significantly influence the intention of adopting hydroponic farming, which was also found to exhibit significant influence on its actual adoption. This study presented valuable insights that contribute to the development of a framework that promotes effective farming practices and strategies. The obtained findings can benefit marketers, practitioners, and policymakers in the agricultural and environmental fields, particularly in their efforts of planning, designing, marketing, and strategizing to promote modern agricultural practices and create a favourable environment for urban hydroponic farming in developing countries.

## 1 Introduction

The rising global population has inadvertently led to the increase in demands for food and other necessities. Moreover, the uncontrollable expansion of cities has led to the significant decrease in cultivable lands for agriculture. After all, the capacity of a country to attain self-sufficiency in food and other basic essentials is reflected through agriculture [1]. It is not sustainable to solely rely on the conventional agricultural production to address food security, which highlights the promising prospects of urban agriculture [2,3].

Urban agriculture has been acknowledged as a feasible solution to the global food insecurity, particularly since the COVID-19 pandemic that has abruptly and adversely affected the urban food supply [4]. According to the World Bank Report Intrado [5], the hydroponics industry was valued at approximately USD 9.5 billion in 2020 and predicted to increase up to USD 22.2 billion by 2028 at a compound annual growth rate (CAGR) of 11.3%. Since 2017, hydroponics projects have been introduced under the United Nations World Food Programme in Algeria, Peru, Jordan, and Zambia as a viable approach of reducing their ongoing food insecurity [6].

Considering the limited water reserves and shrinking land areas, more countries have begun to explore and expand urban hydroponic farming practices and techniques [7]. Apart from their significant prospects for sustainable agriculture, advanced hydroponic systems are generally more environmentally beneficial than conventional soil-based farming [8,9]. Numerous developed and developing countries have made efforts of developing hydroponic farming as a significant priority to regenerate depleted soil resources and prevent further deterioration of soil system [10].

In order to produce healthy crops with excellent nutritional quality and organoleptic attributes, hydroponic farming does not require soil and involves the use of light (as energy), fertilizer, and mineral-enriched water [11]. The capacity of hydroponic farming to reuse water and its nutrients makes it independent of environmental variations, enables effective prevention of soil-borne plant diseases, reduces production costs without compromising its production, and, most importantly, minimises the adverse effects of conventional farming [7,12]. Additionally, hydroponic crops offer superior food value and require considerably less time to harvest [13]. Unlike conventional farming, hydroponic farming requires lower operational and labour costs but requires high initial capital [9]. However, its high initial capital is a major drawback. Nonetheless, hydroponic farming offers significant benefits that it has been adopted on a small scale in numerous major cities as a sustainable food production method [14]. Addressing the need to identify an alternative solution to food insecurity, it is imperative to explore factors that influence urban farming perception, acceptability, and actual adoption of hydroponic farming across diverse cultural and environmental contexts.

The substantial rise of population, climate change, and soil degradation have critically threatened food security in China, reflecting the importance of urban farming in this country [4]. Furthermore, considering the significant number and size of densely populated megacities in China, hydroponic farming, which is more cost- and time-effective and does not require much space, clearly offers a more practical option for the development of urban agriculture [15,16]. At present, hydroponic farming among urbanites in China is still in its initial stages. Moreover, the adoption of hydroponic farming among Chinese urbanites has remained underexplored. Addressing the research gaps in existing literature, the current study focused on exploring the current trend of urban hydroponic farming and perception of hydroponic farming and identifying factors that influence the intention and adoption of hydroponic farming among Chinese urbanites.

In particular, this study examined the influence of tolerance of diversity, innovativeness, knowledge about hydroponic system, attitude towards hydroponic farming, perceived need for hydroponic farming, social influences, and hydroponic farming compatibility on the intention and actual adoption of hydroponic farming among Chinese urbanites. Theory of planned behaviour (TPB) and knowledge-attitude-behaviour (KAB) theory were adapted and integrated for the construction of a holistic framework in this study to examine the interactions of these constructs. Empirical evidence on urbanites’ intention and actual adoption of hydroponic farming can substantially benefit the fields of modern agricultural practices, technologies, and products.

### 1.1 Theoretical foundation

TPB has been considered in numerous studies on the assessment of attitude and intention due to the significance of intention in predicting and influencing the behaviour [[17], [18], [19]]. However, attitude, normative beliefs, and perceived behavioural control in TPB do not adequately explain the determinants of one’s intention to adopt [18,20] Therefore, numerous studies have recommended integrating TPB with other theories or models to comprehensively explore factors that determine one’s intention [21,22].

For instance, Paek et al. [23] and Liu et al. [15] proposed combining TPB and KAB theory. KAB theory assesses the function of knowledge and elucidates the relationship between knowledge and attitude [15]. The KAB theory postulates that incremental and step-by-step improvements in knowledge and attitude stimulate the change in intention or behaviour [24]. In other words, the more knowledge one gains about a certain subject, the more favourable their attitude towards the subject—this consequently drives the actual adoption [ [25,26]]. The KAB theory has been widely applied in various domains, such as information security, healthcare, environment, education, and clinical services [25,27]. However, its applications in modern farming and agricultural technology domain have remained scarce. The current study identified a knowledge gap on hydroponic farming among Chinese urbanites. Thus, the KAB theory was deemed fitting for the current study to adapt in order to gain better insights on the influence of knowledge on attitude.

Besides that, the current study considered four other factors, namely tolerance of diversity, innovativeness, perceived need for hydroponic farming, and hydroponic farming compatibility. These factors were regarded as strong motivational components in the current study’s framework. Prior studies on various agricultural methods, techniques, and products highlighted the significance of these factors in influencing farmers’ and consumers’ intention and actual adoption [[28], [29], [30]].

### 1.2 Development of hypotheses

#### 1.2.1 Tolerance of diversity (TOD)

The ability to embrace various beliefs and traditions that are different from one’s customs reflects tolerance of diversity [29]. Tolerance of diversity drives one’s attitude towards the adoption of diverse traits in socialisation [31]. In the context of agriculture, tolerance of diversity refers to farmers’ ability to adapt to various factors, such as a diverse agro-cultural community [32], agro-biodiversity and food-culture diversity [33], and environmental resource diversity [34]. Accordingly, it is rather improbable to expect novices in farming to instantly adapt in communities given their relatively low tolerance of diversity [32]. Ince [33] examined the African indigenous vegetable farming in urban and peri-urban contexts and found the need to support the diversification of agro-biodiversity, nutrition, and food-culture diversity within both contexts. Besides that, financial diversity and effort diversity, such as socioeconomic background and lifestyle practices, were highlighted as important determinants of urban farming development [34]. In another study, Asravor [35] demonstrated the substantial influence of one’s attitude towards risk-tolerance, such as financial risk and risk related to production volume on the willingness to tolerate diversification in agribusiness. Thus, following hypothesis was proposed for testing in the current study.

H1 Tolerance of diversity positively influences attitude towards hydroponic farming.

#### 1.2.2 Innovativeness (INO)

Innovativeness describes the extent of transformation induced by different factors, such as novel meanings, resources, contexts, and competencies, in contrast to the conventional practices [36]. Miličić et al. [28] found that innovativeness was strongly and significantly related to positive attitude towards aquaponics among Europeans. On a similar note, Hwang et al. [37] highlighted the significant role of perceived innovativeness in forming favourable attitude. Meanwhile, Sanyé-Mengual et al. [38] described innovative practices in urban agriculture as socially, environmentally, and economically novel farming techniques. In a more recent study, Ghandar et al. [39] described farmers’ willingness to adopt innovative practices in agriculture, such as decision analytics, IoT interface, and even remote sensor systems. In fact, agriculture has always been at the leading edge of innovation, and the formation of attitude towards the application of novel technology in farming has consistently demonstrated favourable outcomes [40]. Based on the review of related literature, the following hypothesis was proposed for testing.

H2 Innovativeness positively influences attitude towards hydroponic farming.

#### 1.2.3 Knowledge about hydroponic system (KNO)

Knowledge about hydroponic system refers to information and resources that can enhance farming production to sustain food supply [41]. Such knowledge can significantly improve farming management skills, which include farm management concepts, farming resource maintenance, and farming diversification [42]. The knowledge domain within the context of farming is linked to several social aspects that promote the adoption of sustainable farming practices [43]. The current study viewed knowledge about hydroponic system as the ability to interpret related information, concepts, and norms of hydroponic farming system. Individuals with the knowledge about hydroponic system, such as the features and procedures required, are generally more willing to adopt hydroponic farming [44]. Yano et al. [45] found that Japanese with more knowledge about the artificial light hydroponic system demonstrated favourable attitude towards hydroponic farming, suggesting the significance of knowledge in forming positive attitude. However, Miličić et al. [28] found generally positive attitude towards aquaponics among Europeans in different regions but no significant differences between those with knowledge about aquaponics and those who learned about aquaponics for the first time through the survey [28]. Thus, the following hypotheses were formulated for testing in the current study.

H3 Knowledge about hydroponic system positively influences attitude towards hydroponic farming.

H4 Knowledge about hydroponic system positively influences intention of adopting hydroponic farming.

#### 1.2.4 Attitude towards hydroponic farming (ATH)

Ajzen [17] described attitude as how favourable (or unfavourable) one evaluates a certain behaviour and responses towards the behaviour. Attitude consists of positive and negative attributes, which result in different resultant outcomes [46,47]. Specht [2] reported that only 23% of the participating city dwellers in Berlin preferred to purchase organic foods although the majority of these respondents (87%) demonstrated favourable attitude towards resource-oriented organic urban agriculture. In another similar study, Miličić et al. [28] reported that 38% of the participating respondents across different European nations demonstrated favourable attitude towards aquaponics and preferred aquaponic fishes over traditionally raised fishes, with 23% of them expressed their willingness to pay extra for aquaponics. These findings suggest that having positive attitude does not necessarily translate to actual adoption or behaviour, as it varies according to attributes and circumstances (e.g., aquaponics versus hydroponic system) [28]. In view of the above, the following hypothesis was formulated for testing.

H5 Attitude towards hydroponic farming positively influences intention of adopting hydroponic farming.

#### 1.2.5 Perceived need for hydroponic farming (PEN)

In most cases, perceived need reflects subjective information about the criteria of deciding whether to adopt or participate in a certain activity or behaviour [48]. Prior studies identified perceived need as an important factor that influences intention [48,49]. Individuals must comprehend and recognise whether the solution they select meets their criteria and objectives [50]. For example, farmers in waterlogged lands and flood-prone regions recognise the need to adopt hydroponic farming to produce crops throughout the monsoon floods and to cope with environmental fluctuations [13]. Wiskerke [51] argued on the production biasness in urban food supply and further elaborated the perceived need of urbanites to double the food production based on the belief on the recent increase in food consumption. The intentions to adopt urban and peri-urban farming practices and to include sustainable diets in urban food systems reflect the perceived needs to address issues pertaining to the available cultivable land and to protect the existing agricultural lands from the adverse implications of uncontrollable urbanisation [52]. With that, the current study proposed and tested the following hypothesis.

H6 Perceived need for hydroponic farming positively influences intention of adopting hydroponic farming.

#### 1.2.6 Hydroponic farming compatibility (COM)

Compatibility refers to the extent of an innovation match the current values, needs, and prior experiences of potential adopters [53]. When a new technology is found more beneficial and useful than the prior technology, the compatibility of this new technology increases users’ intention to use the technology [54]. The current study defined hydroponic farming compatibility as the extent to which hydroponic farming can offer greater benefits than the conventional soil-based farming in urban areas with limited cultivable land. Based on a survey conducted across European nations, Miličić et al. [28] identified those who appreciated organic and local products as the most potential users of aquaponic products, which demonstrated their belief on the perceived benefits of aquaponic products over organic products. In another study that focused on environmentally friendly agriculture, Wu et al. [30] discussed the willingness of farmers to use eco-friendly farming practices when they recognised the compatibility of bio-concentrated liquid fertiliser with their existing products and regular practices. Although hydroponic farming is more beneficial than conventional agriculture, the influence of its compatibility on the intention of adopting hydroponic farming has remained unknown. Reimer et al. [55] identified farmers’ portrayal of incompatibility with the methods as the most significant challenge in introducing agricultural conservation practices, such as buffer-strips/channelled waterways (to reduce water wastage) and conservation tillage/no-till (to prevent soil deterioration). Thus, the current study proposed the following hypothesis for testing.

H7 Hydroponic farming compatibility positively influences intention of adopting hydroponic farming.

#### 1.2.7 Social influence

Social influence reflects the extent to which one is influenced by the expectations, suggestions, and views of the surrounding important individuals, such as family, friends, and colleagues—this construct has been widely used to examine consumers’ motivation, intention, and behaviour [56,57]. More precisely, social influence is linked to certain reference groups [58]; the opinions and behaviours of which affect one’s decision-making [59,60]. Social influence may influence one’s views about a particular subject, which may result in the formation of the actual behaviour, such as trying new technologies [56,57,61]. Salim et al. [62] discussed the effects of social influence on the intention and actual adoption of urban agricultural technology. In a more recent study, Tran-Nam et al. [63] highlighted peer influence, such as the communication frequency and the presence of organic farming neighbours, as key aspects of social influence for the uptake of organic agriculture. Besides that, societal norms are major factors that drive organic farming among farmers who are concerned about the negative environmental implications of the standard farming [58,63]. Considering the connection between the adoption of hydroponic farming and the mitigation of environmental issues and urban food insecurity, the following hypothesis was proposed for testing in this study.

H8 Social influence positively influences intention of adopting hydroponic farming.

#### 1.2.8 Intention and actual adoption of hydroponic farming

The tendency to participate in a certain activity due to one’s internal intention processes drive the actual adoption [64]. In particular, the intention to engage in a specific activity affects whether the engagement materialises or otherwise [65]. This is because humans are generally logical thinkers who intend to achieve a certain objective and then act [66]. When it comes to the context of organic farming, intention refers to a farmer’s objective of eliminating hazardous compounds in the farming system [65]. The current study viewed intention of adopting hydroponic farming as one of the factors that influence urbanites’ willingness to adopt hydroponic farming and their actual adoption of hydroponic farming. Fatemi and Rezaei [65] highlighted the positive and strong relationship between intention of adopting organic agricultural practice and actual adoption of organic agricultural practice. Meanwhile, Kashif et al. [67] identified gaps on the relationship between intention and actual adoption of various organic products. Recognising the contradictory findings on this relationship, the following hypothesis was proposed for testing in this study.

H9 Intention of adopting hydroponic farming positively influences actual adoption of hydroponic farming.

**Fig. 1:**
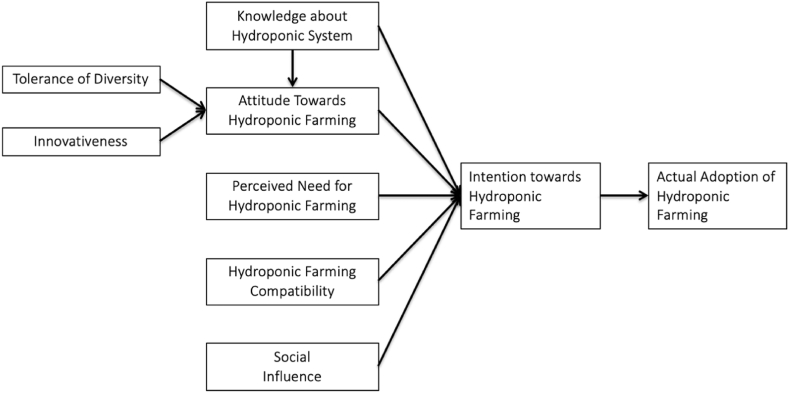
Theoretical framework.

All hypothesised relationships are illustrated in Fig. 1.

## 2 Methodology

The cross-sectional quantitative research design was employed to examine the hypothesised relationships. This study targeted Chinese urbanites of all ages (above 18) and backgrounds. Convenience sampling strategy was applied to select easily reachable and available respondents from the target population [68]. Hair et al. [69] proposed a sample size of 200–400 for PLS-SEM. Referring to the recommendation by Hair et al. [70], G*Power tool was used to determine the minimum sample size for this study. With effect size (*f*^2^) of 0.15, *α*-err prob of 0.05, power (1-*β* err prob) of 0.95, and eight predictors, this study required a minimum sample size of 153 respondents.

### 2.1 Data collection method and procedure

An online survey was conducted from February 2022 to April 2022. The online survey link was shared via WeChat, WenJuanXing, and Questionnaire Star. This study gathered data from 661 respondents to prevent potential complexities of a small sample size of 153 (as calculated via G*Power). The participation of respondents in this survey was entirely voluntary. Furthermore, the confidentiality of the personal information provided by the respondents was ensured.

### 2.2 Measures

To test the proposed hypotheses, this study conducted data collection through a structured questionnaire. The development of the questionnaire survey ensured the use of simple, concise, and unbiased wordings for the respondents to provide their views and responses accurately with ease. All conceptual and measurement items were derived from previous literature reviews and adapted to fit the background of this study and ensure effective reliability and validity. The perceived need for hydroponic farming was adopted from Meadows et al. [71]; Tolerance of diversity was adapted from Cuadrado et al. [72]; Inattentiveness was adapted from Mahmood et al. [73]; Knowledge about hydroponic systems was adapted from Singh & Verma [50], Wang et al. [74], and Dangi et al. [75]; Attitude towards hydroponic farming was adapted from Mahmood et al. [73]; Social influence was adapted from Lin [76] and Rahi et al. [77]; Hydroponics compatibility, intention towards hydroponic farming, and adoption of hydroponic farming were adapted from Yang et al. [78]. All constructs used in this study are presented in ‘Supporting Material 1. Survey Instrument’, were measured using a 7-point Likert scale (1 Strongly Disagree, 7 Strongly Agree). As the online survey targeted respondents in China, the questionnaire survey was constructed in Chinese language. Chinese language experts were appointed to review the accuracy of the intended meaning for each item in the final version of the developed instrument, ensuring accurate responses from the respondents [79]. Complete data as provided as ‘Supporting Material 2. DATA’.

### 2.3 Common method variance

This study selected Harman’s single-factor test to assess the presence of common method variance (CMV) due to its reliability in determining whether CMV significantly affects the study’s model [80]. Based on the obtained results, every single component accounted for 30.00% of the total variation, which did not exceed the maximum threshold of 50%, as recommended by Podsakoff et al. [81]. In other words, the current study’s data did not encounter any CMV issues.

In addition, this study performed Kock’s full collinearity test to assess common method biasness. According to Kock [82], all constructs’ variance inflation factor (VIF) values should not exceed the maximum threshold value of 3.3 to ensure no collinearity issue in the dataset. Referring to Table 1, the recorded VIF values of all constructs in this study ranged from 1.890 to 3.078, which satisfied the threshold criterion. Thus, the single-source data were not skewed and had no collinearity issue.

**Table 1:** Full collinearity test.

Constructs	TOD	INO	PEN	KNO	ATH	SOI	COM	ITO	ADT
Variance Inflation Factor	1.890	3.078	2.013	2.469	2.303	2.156	2.327	3.074	2.100

**Note:** TOD: Tolerance of Diversity; INO: Innovativeness; PEN: Perceived Need for Hydroponic Farming; KNO: Knowledge about Hydroponic Systems; ATH: Attitude towards Hydroponic Farming; SOI: Social Influence; COM: Hydroponics Compatibility; ITO: Intention towards Hydroponic Farming; ADT: Adoption of Hydroponic Farming.

### 2.4 Multivariate normality

This study utilised a statistical web application, specifically Web Power (Link: https://webpower.psychstat.org/wiki/tools/index), to evaluate multivariate normality in terms of “skewness and kurtosis”. Both p-values did not exceed the threshold value of 0.05, as recommended by Cain et al. [83]. These results demonstrated that the current study’s data were not normally distributed.

### 2.5 Data analysis method

As a non-parametric and multivariate approach, PLS-SEM is commonly performed to examine path correlations between latent components [70]. Considering the non-normality issue with the data, the complexity of the research framework and the involvement of multiple elements at various levels, PLS-SEM was deemed fitting, as recommended by Hair et al. [70]. Thus, Partial least squares-structural equation modelling (PLS-SEM) was performed using SmartPLS (V.3.3.5) software. This study also included multi-group analysis, depending on whether or not training was received, which would be better managed through PLS-SEM. Thus, SmartPLS was considered as the most appropriate statistical tool for the current study.

## 3 Results

### 3.1 Demographic characteristics

Table 2 presents the demographic profile of respondents in this study. In particular, the majority of the respondents were female (54.9%). The remaining respondents were male (45.1%). Most of them were in the age group of between 31 and 40 years (33.4%), followed by the age group of between 18 and 30 years (28.7%) and the age group of between 41 and 50 years (21.8%). Besides that, a large proportion of the respondents had a Bachelor’s degree (42.2%), followed by Diploma holders (24.1%). When it comes to monthly income, most of the respondents reported earnings from RM 2,501 to RM 5,000 per month (29.3%). About 27.4% of the total respondents reported earnings from RM 5,001 to RM 10,000. In addition, the majority of respondents (36.3%) indicated living in Central China, followed by East China (29.7%) and Southern China (17.2%). Furthermore, 61.4% of the total respondents received agriculture-related training. However, 59.9% of them received very minimal training (i.e., less than two training sessions). About 20.3% of them received three to five training sessions. Meanwhile, 33.4% of the total respondents gained knowledge through work instruction methods, whereas the other 22.4% gained knowledge about hydroponic agriculture through lecture methods.

**Table 2:** Demographic profile of respondents.

	n	%		n	%
*Gender*			*Monthly income*		
Male	298	45.1	Below CNY 2500	107	16.2
Female	363	54.9	CNY 2501-5000	194	29.3
Total	661	100.0	CNY 5001-7500	181	27.4
			CNY 7501–10,000	98	14.8
*Age*			CNY 10,001–12,500	39	5.9
18–30 years	190	28.7	Above CNY 12,501	42	6.4
31–40 years	221	33.4	Total	661	100
41–50 years	144	21.8			
51–60 years	88	13.3	*Training received*		
Above 60 years	18	2.7	Yes	406	61.4
Total	661	100	No	255	38.6
			Total	661	100
*Location*					
East China	196	29.7	*Times of training*		
Southern China	114	17.2	0-2 times	396	59.9
Central China	240	36.3	3-5 times	134	20.3
North China	47	7.1	6-8 times	71	10.7
Northwest China	20	3	8-10 times	43	6.5
Southwest China	28	4.2	more than 10 times	17	2.6
Northeast China	9	1.4	Total	661	100
Others	7	1.1			
Total	661	100	*Ways to gain knowledge*		
			Lecture method	148	22.4
*Education*			Work instruction method	221	33.4
Secondary school certificate	126	19.1	Game method	93	14.1
Diploma/Technical school certificate	159	24.1	Audio-visual technology method	125	18.9
Bachelor degree or equivalent	266	40.2	Other methods	74	11.2
Master degree	100	15.1	Total	661	100
Doctoral degree	10	1.5			
Total	661	100			

### 3.2 Measurement model (outer model)

According to Hair et al. [70], the measurement model is assessed prior to the assessment of structural model. As for the current study, the internal consistency and reliability, convergent validity, and discriminant validity of the measurement model were assessed to ensure its robustness.

#### 3.2.1 Internal consistency and convergent validity

The reliability and validity of all measures were first evaluated to assess the accuracy of the instrument and the consistency of the measurement structure [69]. Accordingly, Cronbach’s alpha, Dijkstra-Henseler’s rho, composite reliability, and average variance extracted (AVE) and are widely used to determine the internal consistency and convergent validity of constructs. As shown in Table 3, the values of Cronbach’s alpha ranged from 0.888 to 0.927; the values of Dijkstra-Henseler’s rho ranged from 0.890 to 0.928; and the values of composite reliability ranged from 0.923 to 0.948. Values exceeding the threshold value of 0.70 indicate significant internal consistency and reliability [84]. All reported values clearly exceeded 0.70, which confirmed the significant internal consistency and reliability of the model. Meanwhile, the analysis involving AVE calculates the proportion of variance in the constructs that can be explained by latent variables, which reflect the convergent validity of the model and its elements [70]. According to Hair et al. [70], AVE values should exceed 0.50. As shown in Table 3, the recorded values of AVE ranged from 0.708 to 0.821, suggesting significant convergent validity.

**Table 3:** Reliability and validity.

Variables	Items	Mean	Standard Deviation	Cronbach's alpha	Dijkstra-Henseler’s rho	Composite Reliability	Average Variance Extracted
TOD	4	5.1815	1.12072	0.888	0.890	0.923	0.749
INO	5	5.2499	1.05002	0.898	0.899	0.924	0.710
PEN	5	5.1570	1.12259	0.897	0.902	0.924	0.708
KNO	5	5.2242	1.10712	0.921	0.921	0.940	0.760
ATH	5	5.1831	1.08050	0.922	0.922	0.941	0.762
SOI	5	5.1035	1.12533	0.922	0.923	0.941	0.761
COM	5	5.0339	1.18206	0.920	0.922	0.940	0.758
ITO	5	5.2372	1.03555	0.926	0.926	0.944	0.772
ADT	4	5.1142	1.15839	0.927	0.928	0.948	0.821

Note: TOD: Tolerance of Diversity; INO: Innovativeness; PEN: Perceived Need for Hydroponic Farming; KNO: Knowledge about Hydroponic Systems; ATH: Attitude towards Hydroponic Farming; SOI: Social Influence; COM: Hydroponics Compatibility; ITO: Intention towards Hydroponic Farming; ADT: Adoption of Hydroponic Farming.

#### 3.2.2 Discriminant validity

Fornell-Larcker criterion, heterotrait-monotrait (HTMT) ratio, and cross-loadings are common indicators of a model’s discriminant validity. When it comes to Fornell-Larcker criterion, the square root of AVE value of a construct should exceed the variances of any other latent variables in the corresponding column and row [70]. As shown in Table 4, all values of Fornell-Larcker criterion exceeded the correlations in the corresponding columns and rows. Meanwhile, the values of HTMT must not exceed 0.90 [85]. Referring to Table 4, the values of HTMT ranged from 0.465 to 0.774.

**Table 4:** Discriminant validity.

	TOD	INO	PEN	KNO	ATH	SOI	COM	ITO	ADT
*Fornell-Larcker Criterion*									
TOD	0.866								
INO	0.622	0.843							
PEN	0.574	0.640	0.841						
KNO	0.536	0.683	0.519	0.872					
ATH	0.536	0.642	0.598	0.516	0.873				
SOI	0.448	0.566	0.479	0.651	0.542	0.872			
COM	0.430	0.583	0.425	0.464	0.543	0.565	0.870		
ITO	0.541	0.689	0.549	0.613	0.603	0.606	0.718	0.879	
ADT	0.502	0.561	0.519	0.506	0.628	0.492	0.576	0.630	0.906
*Heterotrait-Monotrait Ratio*									
TOD	–								
INO	0.695	–							
PEN	0.641	0.710	–						
KNO	0.593	0.752	0.569	–					
ATH	0.590	0.704	0.654	0.560	–				
SOI	0.492	0.621	0.524	0.706	0.587	–			
COM	0.471	0.638	0.465	0.499	0.588	0.611	–		
ITO	0.596	0.756	0.598	0.663	0.652	0.654	0.774	–	
ADT	0.553	0.614	0.565	0.547	0.679	0.530	0.621	0.679	–

Note: TOD: Tolerance of Diversity; INO: Innovativeness; PEN: Perceived Need for Hydroponic Farming; KNO: Knowledge about Hydroponic Systems; ATH: Attitude towards Hydroponic Farming; SOI: Social Influence; COM: Hydroponics Compatibility; ITO: Intention towards Hydroponic Farming; ADT: Adoption of Hydroponic Farming.

Lastly, cross-loadings represent the outer loadings of the model. Loadings exceeding 0.60 reflect strong discriminant validity [86]. Referring to Table 5, all loadings exceeded the recommended threshold value. Thus, these results confirmed the significant discriminant validity.

**Table 5:** Loading and cross loadings.

	TOD	INO	PEN	KNO	ATH	SOI	COM	ITO	ADT
TOD1	0.869	0.529	0.478	0.457	0.500	0.420	0.389	0.472	0.440
TOD2	0.850	0.546	0.519	0.471	0.447	0.361	0.289	0.480	0.432
TOD3	0.893	0.528	0.498	0.475	0.446	0.368	0.382	0.452	0.442
TOD4	0.851	0.550	0.494	0.452	0.459	0.396	0.426	0.468	0.424
INO1	0.557	0.833	0.545	0.578	0.544	0.450	0.435	0.577	0.484
INO2	0.573	0.860	0.579	0.580	0.576	0.506	0.482	0.603	0.497
INO3	0.498	0.862	0.536	0.554	0.549	0.504	0.513	0.563	0.475
INO4	0.507	0.827	0.510	0.573	0.519	0.435	0.450	0.571	0.422
INO5	0.479	0.829	0.522	0.596	0.513	0.486	0.578	0.591	0.485
PEN1	0.415	0.504	0.821	0.413	0.476	0.399	0.372	0.410	0.395
PEN2	0.460	0.497	0.819	0.397	0.451	0.341	0.320	0.413	0.375
PEN3	0.518	0.546	0.866	0.453	0.501	0.410	0.352	0.471	0.457
PEN4	0.495	0.553	0.838	0.451	0.529	0.423	0.373	0.481	0.459
PEN5	0.515	0.581	0.861	0.462	0.547	0.434	0.370	0.519	0.481
KNO1	0.447	0.590	0.453	0.863	0.446	0.559	0.398	0.532	0.425
KNO2	0.498	0.576	0.444	0.847	0.439	0.512	0.343	0.510	0.416
KNO3	0.446	0.591	0.445	0.897	0.453	0.565	0.383	0.525	0.462
KNO4	0.487	0.595	0.468	0.883	0.452	0.586	0.408	0.551	0.448
KNO5	0.458	0.624	0.452	0.867	0.459	0.613	0.484	0.550	0.452
ATH1	0.469	0.537	0.515	0.446	0.872	0.469	0.472	0.535	0.565
ATH2	0.483	0.587	0.537	0.459	0.868	0.474	0.495	0.541	0.575
ATH3	0.438	0.538	0.481	0.430	0.867	0.470	0.489	0.493	0.538
ATH4	0.479	0.582	0.550	0.470	0.903	0.475	0.443	0.516	0.541
ATH5	0.467	0.556	0.525	0.447	0.853	0.476	0.472	0.543	0.522
SOI1	0.379	0.494	0.421	0.584	0.504	0.860	0.464	0.512	0.436
SOI2	0.413	0.530	0.458	0.593	0.484	0.881	0.503	0.542	0.410
SOI3	0.348	0.485	0.386	0.550	0.433	0.876	0.488	0.500	0.404
SOI4	0.390	0.458	0.399	0.550	0.442	0.871	0.482	0.512	0.397
SOI5	0.418	0.498	0.423	0.563	0.496	0.875	0.523	0.570	0.491
COM1	0.335	0.448	0.339	0.358	0.459	0.500	0.878	0.584	0.507
COM2	0.335	0.486	0.334	0.371	0.438	0.480	0.887	0.591	0.498
COM3	0.412	0.500	0.378	0.420	0.529	0.490	0.856	0.634	0.530
COM4	0.421	0.581	0.429	0.468	0.481	0.508	0.855	0.691	0.507
COM5	0.356	0.506	0.359	0.387	0.449	0.477	0.875	0.608	0.460
ITO1	0.454	0.582	0.483	0.527	0.530	0.544	0.638	0.867	0.535
ITO2	0.471	0.626	0.488	0.561	0.513	0.513	0.613	0.887	0.528
ITO3	0.476	0.610	0.484	0.538	0.535	0.546	0.666	0.893	0.544
ITO4	0.467	0.616	0.456	0.534	0.531	0.529	0.630	0.874	0.578
ITO5	0.508	0.593	0.502	0.532	0.538	0.528	0.606	0.872	0.583
ADT1	0.431	0.498	0.456	0.437	0.565	0.448	0.550	0.562	0.895
ADT2	0.459	0.535	0.475	0.475	0.580	0.471	0.573	0.594	0.910
ADT3	0.468	0.513	0.490	0.477	0.569	0.441	0.510	0.576	0.924
ADT4	0.464	0.487	0.458	0.442	0.562	0.421	0.450	0.550	0.895

Note: TOD: Tolerance of Diversity; INO: Innovativeness; PEN: Perceived Need for Hydroponic Farming; KNO: Knowledge about Hydroponic Systems; ATH: Attitude towards Hydroponic Farming; SOI: Social Influence; COM: Hydroponics Compatibility; ITO: Intention towards Hydroponic Farming; ADT: Adoption of Hydroponic Farming.

### 3.3 Structural model (inner model)

According to Hair et al. [70], it is recommended to evaluate path coefficient (beta value, *β*), coefficient of determination (*r*^2^), and effect size (*f*^2^) for the assessment of structural model. Using the bootstrapping approach, the hypothesised relationships in this study were evaluated based on the p-values, t-values, and *β*-values. Lastly, the multi-group analysis was conducted to evaluate the relationship between intention to adopt hydroponic farming and actual adoption of hydroponic farming.

### 3.4 Testing of hypotheses

Hypotheses were evaluated based on p-values, t-values, and *β*-values. The results of analysis are summarised in Table 6. Firstly, the relationship between TOD and ATH was found to be statistically significant and positive (*β*-value = 0.204, t-value = 4.409, p-value = 0.000). Thus, H1 was supported. Similarly, both H2 (*β*-value = 0.445, t-value = 8.573, p-value = 0.000) and H3 (*β*-value = 0.103, t-value = 1.847, p-value = 0.033) were supported, which confirmed the strong and significant influence of INO and KNO on ATH.

**Table 6:** Hypothesis testing.

Hypothesis		Beta	t-value	p-value	Decision
H_1_	TOD → ATH	0.204	4.409	0.000	Supported
H_2_	INO → ATH	0.445	8.573	0.000	Supported
H_3_	KNO → ATH	0.103	1.847	0.033	Supported
H_4_	KNO → ITO	0.222	5.177	0.000	Supported
H_5_	ATH → ITO	0.124	2.451	0.007	Supported
H_6_	PEN → ITO	0.132	3.111	0.001	Supported
H_7_	COM → ITO	0.447	8.871	0.000	Supported
H_8_	SOI → ITO	0.078	1.588	0.056	Rejected
H_9_	ITO → ADT	0.630	12.336	0.000	Supported

**Note:** TOD: Tolerance of Diversity; INO: Innovativeness; KNO: Knowledge about Hydroponic Systems; PEN: Perceived Need for Hydroponic Farming; ATH: Attitude towards Hydroponic Farming; SOI: Social Influence; COM: Hydroponics Compatibility; ITO: Intention towards Hydroponic Farming; ADT: Adoption of Hydroponic Farming.

In the second level of analysis, the obtained results revealed the statistically significant and positive relationship between KNO and ITO (*β*-value = 0.222, t-value = 5.177, p-value = 0.000). Thus, H4 was accepted. Besides that, ATH (*β*-value = 0.124, t-value = 2.451, p-value = 0.007), PEN (*β*-value = 0.132, t-value = 3.111, p-value = 0.001), and COM (*β*-value = 0.447, t-value = 8.871, p-value = 0.000) exhibited statistically significant and positive influence on ITO. In other words, H5, H6, and H7 were supported. On the other hand, the results demonstrated the positive influence of SOI (*β*-value = 0.078, t-value = 1.588, p-value = 0.056) on ITO, but the relationship was found to be statistically insignificant. This study obtained inadequate evidence to support H8. Lastly, ITO was found to have statistically significant influence on ADT (*β*-value = 0.630, t-value = 12.336, p-value = 0.000). Thus, H9 was supported.

#### 3.4.1 Coefficient of determination (r^2^)

Coefficient of determination (*r*^2^) is a measure of the extent of explained variances or the proportion of the variation in the dependent variable that is explained by a linear model. According to Hair et al. [84], endogenous variables with *r*^2^ of 0.75, 0.50, or 0.25 are classified as having significant, moderate, or poor explanatory power, respectively. Referring to Table 7, ATH recorded *r*^2^ of 0.448, suggesting that TOD, INO, and KNO can explain 44.8% of the total variation in ATH. Besides that, ITO recorded *r*^2^ of 0.652. These results indicated that PEN, ATH, SOI, and COM can explain 65.2% of the total variation in INO, confirming moderate explanatory power. Lastly, ADT recorded *r*^2^ of 0.397, indicating that INO can explain 39.7% of the total variation in ADT.

**Table 7:** Coefficient of determination (*r*^*2*^*)*.

Variables	R Square Adjusted
Attitude towards Hydroponic Farming	0.448
Intention towards Hydroponic Farming	0.652
Adoption of Hydroponic Farming	0.397

**Note:***r*^*2*^ value interpretation (≥0.75- Strong,≥0.50- moderate,≥0.25- Weak) [84].

#### 3.4.2 Effect size (f^2^)

Effect size (*f*^2^) determines whether each specific independent latent construct and dependent latent construct exhibit functional impact [87]. According to Cohen [87], effect size can be trivial (<0.02), minor (≥0.02), medium (≥0.15), or substantial (≥0.35). Considering the research framework and different features of elements, it can be challenging to determine the appropriateness of the basic recommendations in achieving substantial effect size [69]. Referring to Table 8, the obtained results revealed minor effect size for the following: (1) TOD → ATH (*f*^2^ = 0.045); (2) PEN → ITO (*f*^2^ = 0.029); (3) KNO → ITO (*f*^2^ = 0.072); (4) ATH → ITO (*f*^2^ = 0.022). Meanwhile, the following paths demonstrated medium effect size: (1) INO → ATH (*f*^2^ = 0.158); (2) COM → ITO (*f*^2^ = 0.343). Lastly, the effect size in the relationship between ITO and ADT (ITO → ADT; *f*^2^ = 0.01) was found substantial.

**Table 8:** Effect size.

Associations	*f*^*2*^	Effect Size
TOD → ATH	0.045	Small
INO → ATH	0.158	Medium
KNO → ATH	0.010	Trivial
KNO → ITO	0.029	Small
ATH → ITO	0.072	Small
PEN → ITO	0.022	Small
COM → ITO	0.008	Trivial
SOI → ITO	0.343	Medium
ITO → ADT	0.659	Substantial

**Note1:** TOD: Tolerance of Diversity; INO: Innovativeness; PEN: Perceived Need for Hydroponic Farming; KNO: Knowledge about Hydroponic Systems; ATH: Attitude towards Hydroponic Farming; SOI: Social Influence; COM: Hydroponics Compatibility; ITO: Intention towards Hydroponic Farming; ADT: Adoption of Hydroponic Farming.

**Note2:** f^2^ score interpretation (≥0.35- substantial effect size,≥0.15– medium effect size,≥0.02- small effect size and <0.02- trivial effect size) [87].

#### 3.4.3 Predictive relevance (Q^2^)

*Q*^2^ test was performed for this study to examine the predictive relevance of endogenous variables, specifically to determine whether exogenous variables have better predictive power than endogenous variables [88]. Accordingly, *Q*^2^ must exceed zero [70]. The obtained results in Table 9 revealed that all endogenous variables recorded *Q*^2^ of more than zero. In other words, the current study’s model demonstrated good predictive relevance and prognostication potential.

**Table 9:** Predictive relevance.

Variables	Q^2^
Attitude towards Hydroponic Farming	0.337
Intention towards Hydroponic Farming	0.498
Adoption of Hydroponic Farming	0.323

**Note:** Q^2^ > 0 is significant [70].

### 3.5 Multi-group analysis

The study proceeded to perform multi-group analysis (MGA) for a more comprehensive interpretation of the results. PLS-MGA is one of the most recommended approaches to assess moderation across several associations, specifically in analysing subgroup heterogeneity [70]. As for the current study, measurement invariance of composite models (MICOM) approach was performed prior to PLS-MGA in order to determine the degree of homogeneity between two groups.

All respondents were classified into two groups: (1) those who received training related to hydroponic farming (N = 406); (2) those who did not receive training related to hydroponic farming (N = 255). The study found that the permutation p-values of all constructs exceeded 0.05, which confirmed the presence of measurement invariances among the analyzed groups. Therefore, path coefficient values were examined through PLS-MGA. As shown in Table 10, the obtained results demonstrated that all p-values for group differences exceeded 0.05. Thus, the data of these two groups exhibited no statistically significant differences in any associations.

**Table 10:** Multi-group Analysis (Training received).

Associations	Yes (N = 406)		No (N = 255)		Difference		Decision
	Beta	*p-*value	Beta	*p-*value	Beta	*p-*value	
TOD- > ATH	0.189	0.001	0.213	0.002	−0.024	0.410	No Difference
INO- > ATH	0.445	0.000	0.470	0.000	−0.024	0.406	No Difference
KNO- > ATH	0.157	0.016	0.000	0.499	0.157	0.086	No Difference
PEN- > ITO	0.172	0.004	0.091	0.058	0.081	0.175	No Difference
KNO- > ITO	0.198	0.000	0.245	0.000	−0.048	0.294	No Difference
ATH- > ITO	0.096	0.086	0.139	0.037	−0.044	0.341	No Difference
SOI- > ITO	0.061	0.133	0.137	0.074	−0.076	0.247	No Difference
COM- > ITO	0.478	0.000	0.383	0.000	0.094	0.200	No Difference
ITO- > ADT	0.620	0.000	0.650	0.000	−0.031	0.379	No Difference

Note: TOD: Tolerance of Diversity; INO: Innovativeness; PEN: Perceived Need for Hydroponic Farming; KNO: Knowledge about Hydroponic Systems; ATH: Attitude towards Hydroponic Farming; SOI: Social Influence; COM: Hydroponics Compatibility; ITO: Intention towards Hydroponic Farming; ADT: Adoption of Hydroponic Farming.

## 4 Discussion

The first level of analysis in this study involved assessing the influence of TOD, INO, and KNO on ATH, whereas the second level of analysis involved assessing the influence of PEN, KNO, ATH, SOI, and COM on ITO. Furthermore, this study examined the relationship between ITO and ADT. Based on the results of PLS-SEM, all relationships, except for the relationship between SOI and ITO, were found to be statistically significant and positive. This section discusses how the obtained findings can be interpreted within the context of a developing country like China.

Firstly, this study empirically proved the significant and positive influence of TOD on ATH. In other words, Chinese urbanites can adapt to any changes and demonstrate favourable attitude towards any types of diversity. This can be attributed to the frequent occurrence of major environmental and catastrophic events (such as heatwaves, droughts, wildfires, floods, hurricanes and sea-level rise) that have substantially changed their lives and inadvertently propelled them to adapt for survival. Another plausible rationalisation lies in the fact that urban areas in China have become increasingly crowded across regions with extremely diverse food culture. Consequently, hydroponic farming has become a feasible alternative for Chinese urbanites with the preference to grow their own regional crop.

Secondly, this study empirically proved the significant relationship between INO and ATH, which supported the findings reported by Miličić et al. [28]. When new technologies and innovations are involved, China has demonstrated how they are often ahead of the curve and an openness to new products and procedures. Likewise, new technologies for agriculture have been introduced and adopted in China. Based on the current study’s results, it was deemed plausible that Chinese urbanites are generally enthusiastic and motivated to try new agricultural methods, such as hydroponic farming. After all, this provides them the sense of satisfaction for keeping up with the modern world and its transformation.

Thirdly, this study demonstrated the significant influence of KNO on both ATH and ITO, which corroborated the findings reported by Ezni et al. [44]. Knowledge about hydroponic system is an important factor that can influence the attitude and intention of Chinese urbanites towards adopting such farming methods. The results of multi-group analysis further revealed no significant differences between those who received training related to hydroponic farming and those who did not receive training related to hydroponic farming. This may be attributed to the growing popularity of hydroponic farming that stimulates the curiosity and interest of nearly everyone. Furthermore, the lockdown period during the COVID-19 pandemic may have motivated many to explore the practice of cultivating plants in urban areas with limited cultivable lands. Consequently, they may have gained considerable knowledge about hydroponic farming and its benefits that meet their needs.

Besides that, this study demonstrated the significant influence of PEN on ITO, suggesting the significance of hydroponic farming in satisfying various needs of Chinese urbanites—for examples, food security and nutritional security for the growing populations, catastrophic events, uncontrollable expansion of cities that results in limited cultivable lands, soil quality degradation due to chemical and other waste, and the high cost of recycling wastewater. These various circumstances have contributed to the growing interest in hydroponic farming among Chinese urbanites for a feasible approach that allows soil-less farming with minimal environmental impact and optimum water utilisation.

This study also demonstrated the significant influence of COM on ITO, which supported the findings reported by Wu et al. [30]. This may be attributed to the soil-less concept of hydroponic farming that is not substantially influenced by environmental variations. It may be readily available as vertical farming. Furthermore, hydroponic farming is easily maintained with fewer cases of pests and diseases and does not involve soil-borne dirt, which are more beneficial in urban settings than other conventional farming methods.

Surprisingly, this study found that SOI exhibited no significant influence on ITO. However, Tran-Nam et al. [63] reported otherwise. This may be due to the development of hydroponic farming, which is still in its early stages. Most individuals only experiment with hydroponic farming and have inadequate expertise to recommend this practice to others. Besides that, the adoption of hydroponic farming requires major initial investment, which can be a challenge for those with limited financial resources. Moreover, the poor distribution of reliable information about the procedures, production volume, and crop variations creates communication gap among the members of communities.

Lastly, the current study demonstrated the significant and positive influence of ITO on ADT. Fatemi and Rezaei [65] reported similar findings. This implies the promising prospects of motivating Chinese urbanites to adopt hydroponic farming by instilling an aspiration in them. Additionally, food insecurity and catastrophic events have propelled the willingness of Chinese urbanites to consider adopting hydroponic farming. Considering the current circumstances, hydroponic farming serves as a practical means that fulfils various needs, such as food security.

## 5 Implications

### 5.1 Theoretical implications

This study presented significant theoretical implications within the context of urban agriculture and hydroponic farming in China through the combination of two prominent theories, specifically TPB and KAB theory, with the inclusion of four additional factors, namely TOD, INO, PEN, and COM. The current study served as the first to incorporate all these factors within a single framework to explore urban agriculture in cities of China. As previously discussed, KNO was found to have significant influence on both ITO and ATH. Simultaneously, both TOD and INO had substantial influence on ATH. These results validated the robustness of the proposed model framework and demonstrated the feasibility of integrating KAB and TPB within the context of hydroponic farming in China. PEN and COM exhibited significant influence on ITO, which ensured the adequacy of TPB with the addition of more compelling factors in elucidating the current study’s context. Last but not least, the explanatory power of ITO achieved 65.2%, which indicated the appropriateness of incorporating the additional factors into TPB and KAB in predicting intention.

### 5.2 Practical implications

This study also presented significant practical implications to the field of agriculture in relation to the country’s economic growth. Furthermore, the constructs and perspectives considered in this study were deemed fitting for the development of various types of agriculture, not limited to hydroponic farming—for examples, aquaponics and organic farming. Focusing on the expansion of urban agriculture in China, Liu et al. [15] highlighted the minimal business interest towards urban agriculture as its economic benefits are perceived less substantial in this country. Thus, the obtained findings of the current study were expected to substantially benefit practitioners and industry players in their efforts of developing more successful business strategies. Besides that, the current study observed the promising prospects of Chinese urbanites’ innovativeness and openness to innovative farming technologies, such as vertical farming and crop variations. Based on the findings of the current study on the influence of knowledge about hydroponic farming, local authorities and other relevant stakeholders should consider organising more practical and long-term training initiatives to expose the urban communities to knowledge about agricultural technologies and equip hydroponic system suppliers with better knowledge and skills. Moreover, considering the significant compatibility and benefits of hydroponic farming, as compared to the conventional soil-based farming, agricultural specialists and researchers can take on an active role of developing and enhancing the features and processes of the hydroponic system for everyone to adopt this practice with ease. Apart from that, the rising population growth, rapid urbanisation, and food security and other environmental issues have contributed to higher perceived need for hydroponic farming, especially in urban areas. Therefore, various initiatives related to conservation agricultural practices should be considered on a larger scale. Surprisingly, the current study found that social influence had no significant effect, which subsequently highlighted the significance of raising public awareness through effective advertising, dissemination of knowledge, and promotion of success stories of hydroponic entrepreneurs. Last but not least, policymakers and regulators should consider highlighting the significant features of hydroponic farming, such as the nutritional advantages of hydroponic foods, the opportunities of being self-employed, the benefits of securing food for own consumption, and exclusive earning opportunities through hydroponic farming, to inspire urban communities to adopt hydroponic farming.

## 6 Conclusion

Following the rising population growth and uncontrollable urbanisation, the increasing demand for food supply has highlighted the indispensable need to expand urban agriculture. Hydroponic farming has been acknowledged as a feasible means that promotes more sustainable use of resources and producing high yields without compromising the environment and public health [7]. Using both TPB and KAB theory with the four additional factors, the current study aimed to examine the intention and adoption of hydroponic farming among Chinese urbanites. The analysis of the online survey data gathered from 661 respondents in various major cities in China revealed the significant influence of tolerance of diversity, innovativeness, and knowledge about hydroponic system on the attitude towards hydroponic farming. Furthermore, this study empirically demonstrated the significant influence of knowledge about hydroponic system, attitude towards hydroponic farming, perceived need for hydroponic farming, and hydroponic farming compatibility on the intention to adopt hydroponic farming among Chinese urbanites. Another notable finding was the insignificant influence of social influence on intention, which implied the need for more attention to promote and expand hydroponic farming in urban areas of China. The increasing number of catastrophic events and other major environmental issues has led to the growing awareness about food-borne health hazards and food security, which has subsequently motivated urbanites to consider the adoption of hydroponic farming and other alternative means of urban farming. The current study highlighted significant factors that should be considered in the development of effective policies and strategies to promote the development and expansion of modern agriculture and to provide a more favourable environment for hydroponic farming in China and other developing countries. The obtained findings also assist the Chinese government in the development of rules and regulations for the development of agriculture in urban settings, which can benefit the country’s economic growth, food security, and unemployment issues.

This study encountered several limitations. Firstly, the current study gathered data from a small sample using the conventional sampling strategy, reducing the generalisability of the obtained findings. Therefore, it is recommended for future research on hydroponic farming to consider a larger sample size from a population of diverse demographics across different regions. Secondly, the current study exclusively focused on several components of attitude and intention and overlooked the influence of other key components, such as technology acceptance factors. Therefore, it is recommended for future research to consider the influence of other potential factors to enhance the current understanding on attitude, intention, and actual behaviour. Lastly, the current study adopted a cross-sectional research design. As a result, the evaluation of behaviour was limited over time. Addressing that, it is recommended for future research to utilise a longitudinal approach to examine the long-term effects of the constructs and their relevant associations over time.

## Declarations

### Author(s) contribution

Gao Jingzu and Qing Yang: Conceived and designed the experiments; Contributed reagents, materials, analysis tools or data; Wrote the paper. Abdullah Al Mamun and Farzana Naznen: Conceived and designed the experiments; Analyzed and interpreted the data; Wrote the paper.

### Availability of data and materials

The original contributions presented in the study are included in the article/Supplementary Material, further inquiries can be directed to the corresponding author/s.

### Conflict of interest

The authors declare that the research was conducted in the absence of any commercial or financial relationships that could be construed as a potential conflict of interest.

### Funding

This research received no specific grant from any funding agency in the public, commercial, or not-for-profit sectors.
