# Supplementary material for: Predicting the intention and adoption of hydroponic farming among Chinese urbanites
Source: Heliyon. 2023 Mar 9;9(3):e14420. doi: 10.1016/j.heliyon.2023.e14420 (PMC10010998; doi:10.1016/j.heliyon.2023.e14420)
Supplement: Multimedia component 2 [file mmc2.docx]

**Supporting Material 1.** Survey Instrument

| Code | Question |
| --- | --- |
| TOD1 | I believe that adopting hydroponic farming will be inexpensive. |
| TOD2 | I believe that using hydroponic farming is a good idea in general. |
| TOD3 | I do not believe that hydroponic farming will be difficult to implement. |
| TOD4 | My mental notion of such a hydroponic system would be that hydroponic farming isn't all that difficult. |
| INO1 | I will try to use hydroponic system even if I am not familiar with it. |
| INO2 | I do not feel hesitant to use hydroponic system. |
| INO3 | I am excited to use hydroponic system. |
| INO4 | I am open to experimenting with new things and methods of farming. |
| INO5 | I keep up with the latest technological development in agriculture. |
| PEN1 | Hydroponic systems produce higher yields than traditional urban gardening methods. |
| PEN2 | It takes less time and effort to use a hydroponic system than it does to use traditional urban gardening methods. |
| PEN3 | Hydroponic systems are more beneficial than traditional urban gardening methods. |
| PEN4 | When compared to the vegetables available on the market, hydroponic systems provide healthier options. |
| PEN5 | The use of hydroponic systems can improve the overall quality of life for urban botanists. |
| KNO1 | Even though I am unfamiliar with hydroponic systems, I will attempt to use one. |
| KNO2 | I have no reservations about utilising a hydroponic system. |
| KNO3 | I'm looking forward to using a hydroponic system. |
| KNO4 | I'm willing to try out new ideas and farming techniques. |
| KNO5 | I keep up with the latest agricultural technological advancements. |
| ATH1 | Hydroponic farming is a great alternative to regular farming in my opinion. |
| ATH2 | Using hydroponic farming boosts my chances of producing agricultural products of the expected quality. |
| ATH3 | Increasing agricultural yield through using hydroponic farming is a good idea. |
| ATH4 | There are various advantages to using hydroponic gardening. |
| ATH5 | Hydroponic farming allows you to save time and effort. |
| SOI1 | Hydroponic farming is recommended by urban farmers and community people who have affected me. |
| SOI2 | Hydroponic farming is something that urban farmers and others close to me believe I should consider. |
| SOI3 | Individuals close to me will support my decision to use hydroponic farming. |
| SOI4 | The advantages of employing hydroponic farming were explained to me by urban farmers and society members who impacted me. |
| SOI5 | The process of adopting hydroponic farming was presented to me by urban farmers and society people who inspired me. |
| COM1 | I have all of the resources I need to apply hydroponic farming techniques. |
| COM2 | I have the information required to apply hydroponic farming techniques. |
| COM3 | My hydroponic system is compatible with my urban garden. |
| COM4 | When I'm having trouble using the hydroponic system on my farm, I can seek assistance from others. |
| COM5 | I believe that I have complete control over my hydroponic system. |
| ITO1 | The hydroponic growing method is what I intend to use. |
| ITO2 | In the foreseeable future, I am willing to employ hydroponic farming. |
| ITO3 | In the near future, I will continue to use hydroponic gardening. |
| ITO4 | I will tell people about hydroponic farming. |
| ITO5 | Because I recognise the advantages of hydroponic farming, I aim to use it. |
| ADT1 | Because I want a high-quality produce, I employ hydroponic farming. |
| ADT2 | For maximum efficiency, I employ a hydroponic farming system. |
| ADT3 | To reduce environmental impact, I employ hydroponic farming. |
| ADT4 | Because I am concerned about my health and the environment, I employ hydroponic farming. |

**Note:** TOD: Tolerance of Diversity; INO: Innovativeness; PEN: Perceived Need for Hydroponic Farming; KNO: Knowledge about Hydroponic Systems; ATH: Attitude towards Hydroponic Farming; SOI: Social Influence; COM: Hydroponics Compatibility; ITO: Intention towards Hydroponic Farming; ADT: Adoption of Hydroponic Farming
